# Development, Acceptance, and Concerns Surrounding App-Based Services to Overcome the COVID-19 Outbreak in South Korea: Web-Based Survey Study

**DOI:** 10.2196/29315

**Published:** 2021-07-30

**Authors:** Jihwan Park, Jinhyun Han, Yerin Kim, Mi Jung Rho

**Affiliations:** 1 School of Software Convergence College of Software Convergence Dankook University Yongin-si Republic of Korea; 2 Department of Urology, Seoul St. Mary’s Hospital College of Medicine The Catholic University of Korea Seoul Republic of Korea; 3 Department of Korean Language and Literature The Anyang University of Korea Anyang-si Republic of Korea

**Keywords:** COVID-19, app-based services, acceptance, concerns, epidemiological investigation, self-route management app, privacy

## Abstract

**Background:**

Since the COVID-19 outbreak, South Korea has been engaged in various efforts to overcome the pandemic. One of them is to provide app-based COVID-19–related services to the public. As the pandemic continues, a need for various apps has emerged, including COVID-19 apps that can support activities aimed at overcoming the COVID-19 pandemic.

**Objective:**

We aimed to determine which apps were considered the most necessary according to users and evaluate the current status of the development of COVID-19–related apps in South Korea. We also aimed to determine users’ acceptance and concerns related to using apps to support activities to combat COVID-19.

**Methods:**

We collected data from 1148 users from a web-based survey conducted between November 11 and December 6, 2020. Basic statistical analysis, multiple response analysis, and the Wilcoxon rank sum test were performed using R software. We then manually classified the current status of the development of COVID-19–related apps.

**Results:**

In total, 68.4% (785/1148) of the respondents showed high willingness to protect themselves from COVID-19 by using related apps. Users considered the epidemiological investigation app to be the most necessary app (709/1148, 61.8%) overall, followed by the self-management app for self-isolation (613/1148, 53.4%), self-route management app (605/1148, 52.7%), COVID-19 symptom management app (483/1148, 42.1%), COVID-19–related information provision app (339/1148, 29.5%), and mental health management app (270/1148, 23.5%). Despite the high intention to use these apps, users were also concerned about privacy issues and media exposure. Those who had an underlying disease and had experience using COVID-19–related apps showed significantly higher intentions to use those apps (*P*=.05 and *P*=.01, respectively).

**Conclusions:**

Targeting users is very important in order to design and develop the most necessary apps. Furthermore, to gain the public’s trust and make the apps available to as many people as possible, it is vital to develop diverse apps in which privacy protection is maximized.

## Introduction

### Background

Since the outbreak of COVID-19, countries worldwide have been engaging in various efforts to overcome the challenges associated with it. One of these efforts include providing app-based services, such as COVID-19 contact tracing apps, to support activities aimed at combating COVID-19 [1-4].

South Korea has been integrating digital technology to make it applicable to all fields [5,6], including surveillance, testing, contact tracing, and self-isolation, as well as apps providing COVID-19–related information. Several COVID-19–related apps have been developed and are currently being used, including the Self-Quarantine Safety Protection app [7] and apps for COVID-19 symptom management app and self-isolation. These apps have greatly helped South Korea in responding to the COVID-19 crisis. However, as the COVID-19 pandemic continues, the need for more diverse apps is emerging, such as COVID-19 vaccine apps [8], epidemiological investigation apps, self-route management apps, and mental health management apps.

To support activities aimed at overcoming COVID-19, diverse apps need to be developed for specific purposes. It is also vital to ensure that the majority of people can be assisted through these apps. Therefore, to ensure the effectiveness of COVID-19–related apps, we need to learn more about the apps that people need, as well as their acceptance and concerns regarding using these apps, for example, concerns regarding security issues. However, although security and information protection issues may arise while developing and using these apps [5,9-11], the use of technology during COVID-19 has focused on using larger amounts of personal data to contain the spread of COVID-19 [12], rather than reflecting on users’ intentions or concerns.

For COVID-19–related technologies to be effective, most people need to be able to use them. To achieve this, we need to focus on users’ intentions and concerns, rather than adopting a technical approach [13]. Therefore, in this study, we aimed to determine the most necessary apps as preferred by users and identify the current status of the development of COVID-19–related apps in South Korea. Furthermore, we aimed to determine users’ acceptance and concerns related to using apps to overcome the COVID-19 crisis.

### Current Status of Development of COVID-19–Related Apps in South Korea

We organized various COVID-19–related apps developed in South Korea according to their release date (Table 1). Thus far, these apps can be classified according to the following main function types: (1) COVID-19–related information provision, (2) COVID-19 symptom management, (3) COVID-19 self-diagnosis, (4) self-route management, (5) mapping of COVID-19 cases, and (6) self-report of confirmed COVID-19 cases.

In early 2020, there were many apps providing COVID-19–related information, but over time, these evolved into COVID-19 symptom management and self-route management apps. Informational apps focus on providing information on the current status of COVID-19; subsequently, information relevant to the present state of COVID-19, such as information about masks and vaccines, is gradually updated and modified to remain relevant. For apps related to self-route management and mapping of COVID-19 cases, however, information is automatically saved using GPS or a QR code. Furthermore, these apps feature a function notifying users of the risk rate, such as mapping confirmed persons with COVID-19. Detailed information about the apps can be found in Table S1 of Multimedia Appendix 1.

**Table 1:** COVID-19–related apps and app functions in South Korea.

No.	Release date	App name	Functions of COVID-19–related apps							OS
			Information provision	Symptom management	Self-diagnosis	Self-route management	Mapping of cases	Self-report of confirmed cases		
1	February 2020	CORNANOW	✓						Android	
2	February 7, 2020	Corona Explorer (코로나 탐색기)	✓						Android	
3	February 17, 2020	Corona App (코로나앱)	✓						Android	
4	February 25, 2020	Corona contact test (코로나 접촉검사)					✓		Android	
5	February 26, 2020	Corona 19 situation board (코로나19 상황판)	✓						Android	
6	March 2, 2020	Corona 19 status board (코로나19 현황판)	✓						Android	
7	March 6, 2020	Corona 19 Gyeongnam (코로나19 경남)	✓						Android	
8	March 6, 2020	Corona compass (코로나침반)	✓				✓		Android	
9	March 6, 2020	Corona Map (코로나맵)	✓						Android	
10	March 9, 2020	Wear mask (웨어마스크)	✓						Android	
11	March 9, 2020	Corona 19 news delivery (코로나19 소식전달)	✓						Android	
12	March 10, 2020	Corona pin (코로나핀)	✓						Android	
13	March 11, 2020	Coronaga (코로나가)					✓		Android	
14	March 11, 2020	NEAR	✓						Android	
15	March 12, 2020	Corona Map Wiki (코로나맵위키)	✓						Android	
16	March 12, 2020	Carrot Mask	✓						Android	
17	March 18, 2020	Mark (마크)	✓						Android	
18	March 18, 2020	Coback Plus (코백플러스)	✓						Android	
19	March 20, 2020	Where is the mask (마스크어딨니)	✓						Android	
20	March 20, 2020	Mask time (마스크타임)	✓						Android	
21	March 20, 2020	Let me know (알려줘)	✓						Android	
22	March 30, 2020	Corona 19 self-diagnosis (코로나19 자가진단)			✓				Android	
23	April 2020	BMC Corona 19 employee guardian BMC (코로나19 직원지킴이)		✓					Android, iOS	
24	April 2020	Search for COVID-19 guidelines (코로나19 지침 검색)	✓						Android, iOS	
25	April 6, 2020	Corona World (코로나월드)	✓						Android	
26	May 22, 2020	JINOSYS	✓			✓			Android	
27	June 2020	Incheon Corona 19 freeze (인천 코로나19 꼼작마!)	✓	✓		✓			Android, iOS	
28	June 2, 2020	School safety guard (학교 안전지킴이)		✓					Android	
29	July 13, 2020	Corona Memo (코로나메모)				✓			Android	
30	August 2020	FAMY 2.0				✓			Android	
31	August 7, 2020	Corona index (코로나지수)	✓						Android	
32	August 10, 2020	Corona Pass (코로나패스)				✓			Android	
33	August 31, 2020	Corona detector (코로나 탐지기)					✓		Android	
34	September 8, 2020	KFKOREA	✓						Android	
35	September 11, 2020	Corona location tracking (코로나위치추적)				✓			Android	
36	October 6, 2020	Avoiding corona (코로나피하go)	✓			✓			Android	
37	October 12, 2020	Koala	✓			✓			Android	
38	December 1, 2020	Corona Alert (코로나알리미)	✓						Android	
39	December 11, 2020	COVID SHIELD	✓						Android	
40	December 23, 2020	Corona Safer	✓			✓	✓		Android	
41	January 6, 2021	Hanyang Univ. Corona contact tracking app (코로나 접촉 추적앱)				✓		✓	Android	
42	January 28, 2021	Corona traffic light (코로나 신호등)	✓						Android	
43	February 3, 2021	Corona 19 vaccine reminder (코로나19 백신 알리미)	✓	✓					Android	
44	February 8, 2021	All about the corona status (코현모)	✓						Android	
45	February 9, 2021	Corona traffic safety (코로나 동선 안심이)				✓			Android, iOS	
46	February 16, 2021	Corona magnifier (코로나돋보기)	✓						Android	
47	March 3, 2021	Corona bored (코로나지겹다)	✓						Android	
48	March 22, 2021	Corona vaccine reminder (코브리움)	✓						Android	

## Methods

### Study Sample

We conducted a web-based survey between November 11 and December 6, 2020. The number of confirmed COVID-19 cases during the survey period ranged from 143 (on November 11) to 631 (on December 6). On November 1, 2020, the Korean government announced a plan to reorganize social distancing measures by subdividing social distancing into three to five stages; this came into effect on November 7, 2020. Thus, during the survey period, social distancing levels ranged from stage 1 to stage 2, based on the five stages of social distancing [14].

We had limitations in conducting a survey that included the total Korean population. Therefore, the survey was conducted keeping in mind the cost and time of distributing the questionnaire. In South Korea, as of December 6, 2020, Seoul, Gyeonggi-do, Incheon, and Daegu had the highest number of COVID-19 cases nationwide, accounting for 79% of all COVID-19 cases in South Korea [15].

We posted the survey recruitment notice on bulletin boards of online cafes, such as Korean portal online cafes (NAVER) [16], as well as university and college student community bulletin boards. In addition, a questionnaire was also distributed through referrals from cafe users. A total of 1170 people responded. After duplicate and incorrect responses were excluded, 1148 valid, completed questionnaires were obtained. The survey ended on December 6, where the proportion of survey respondents by region was similar to the proportion of COVID-19 cases by region as of December 6.

### Review of COVID-19–Related Apps and Functions Developed in South Korea

To determine the current status of apps developed in South Korea, we conducted a search on application software downloading services such as the Apple App Store, Google Play Store, and Naver One Store. We aimed to find all COVID-19–related apps developed after January 2020, that is, after the COVID-19 outbreak was reported. We used keywords such as “COVID,” “COVID-19,” “Corona,” “Corona 19,” and “infectious disease.” We excluded COVID-19–related apps developed by the Ministry of the Interior and Safety and the Ministry of Health and Welfare. Thus, we found a total of 54 apps. Among these, overseas apps and apps introduced before the COVID-19 outbreak were excluded. For the remaining 48 apps, two medical informatics professors (JP and MJR) and two researchers (JH and YK) manually organized the app features into categories (described below) over four meetings.

To categorize these apps, it was necessary to largely classify them by app features. However, there was no clear criteria for categorizing the app functions. Based on previous studies [12,17,18], we classified the apps developed in South Korea thus far into the following main function types to determine their current status: (1) COVID-19–related information provision, (2) COVID-19 symptom management, (3) COVID-19 self-diagnosis, (4) self-route management, (5) mapping of COVID-19 cases, and (6) self-report of COVID-19 confirmed cases.

### The Intention to Use COVID-19–Related Apps

We developed a questionnaire determining the intention to use COVID-19–related apps based on previous studies [19,20]. Intention to use is the most frequently used variable in research on technology acceptance and is widely used in the health care field [21,22]. Additionally, the questionnaire items were modified for this study. That is, “intention to use” was defined as the degree to which a user’s behavioral intention indicated their willingness to use COVID-19–related apps. Responses were given on a 5-point Likert scale ranging from 1 = “very unwilling” to 5 = “very willing.”

### Searches of App-Based Services to Support Activities to Combat COVID-19

COVID-19–related apps that were currently deemed as necessary were selected based on the six abovementioned functions. However, we added additional apps, namely the epidemiological investigation app, self-management app for self-isolation, and mental health management app.

Finally, we classified app-based services to support activities to overcome the COVID-19 crisis according to six app types: (1) epidemiological investigation apps, (2) self-management apps for self-isolation, (3) self-route management app, (4) COVID-19 symptom management app, (5) COVID-19–related information provision app, and (6) mental health management app.

### Statistical Analysis

The question asking participants which app services are needed to support activities aimed at overcoming the COVID-19 crisis was a multiple-response question; thus, multiple response analysis was used. The Wilcoxon rank sum test [23] was used to analyze people’s intention to use app-based services required to overcome COVID-19. Basic statistical analysis, multiple response analysis, and Wilcoxon rank sum test were conducted using R software (version 3.6.1). Furthermore, we manually classified the current status of the development of COVID-19–related apps.

### Ethics

The study procedures were carried out in accordance with the Declaration of Helsinki and were approved by the Institutional Review Board of Catholic University (MC20QISI0125). Participants’ data were anonymized to ensure confidentiality was maintained.

## Results

### Participants’ Characteristics

Of the total 1148 respondents, 675 (58.8%) were female and the majority (n=475, 41.4%) were in their 30s (Table 2). The proportion of married respondents was 50.6% (581/1148). Furthermore; 846 (73.7%) of the respondents had a university degree or higher; 592 (51.6%) were employed in professional, managerial, and white-collar jobs; and 128 (11.1%) were medical staff. Moreover, 883 (76.9%) respondents lived in Seoul, Gyeonggi-do, Incheon, and Daegu.

**Table 2:** Demographic characteristics (N=1148).

Characteristic		Participants, n (%)
**Gender**		
	Male	473 (41.2)
	Female	675 (58.8)
**Age**		
	18 and 19	14 (1.2)
	20-29	342 (29.8)
	30-39	475 (41.4)
	40-49	238 (20.7)
	>50	79 (6.9)
**Marital status**		
	Single	551 (48)
	Married	581 (50.6)
	Other (including divorced, separated, or widowed)	16 (1.4)
**Education**		
	High school graduation or lower	93 (8.1)
	College students	209 (18.2)
	University graduation or higher	846 (73.7)
**Occupation**		
	Other or unemployed	56 (4.9)
	Service, sales, or production	96 (8.4)
	Self-employed or freelancer	98 (8.5)
	Office worker, professional, or administrative job	592 (51.6)
	Housewife	133 (11.6)
	Student	173 (15.1)
**Medical profession**		
	No	1020 (88.9)
	Yes	128 (11.1)
**Salary (US $)^a^**		
	<1825.82	73 (6.4)
	1825.82-3,651.63	426 (37.1)
	3,651.63-5,477.45	330 (28.7)
	>5,477.45	319 (27.8)
**Location**		
	Seoul	420 (36.6)
	Gyeonggi-do	299 (26)
	Daegu Metropolitan City	103 (9)
	Incheon Metropolitan City	61 (5.3)
	Daejeon	57 (5)
	Busan	51 (4.4)
	Gyeongsangbuk-do	29 (2.5)
	Chungcheongnam-do	25 (2.2)
	Gwangju	21 (1.8)
	Ulsan Metropolitan City	17 (1.5)
	Gyeongsangnam-do	15 (1.3)
	Gangwon-do	13 (1.1)
	Jeollabuk do	13 (1.1)
	Chung-cheong bukdo	10 (0.9)
	Sejong City	7 (0.6)
	Jeju Special Self-Governing Province	4 (0.3)
	Jeollanam-do	3 (0.3)

^a^A currency exchange rate of US $1= ₩1095.40 is applicable (buy and sell base rate on January 13, 2021).

### COVID-19–Related Characteristics

Among the 1148 respondents, 95 (8.3%) had an underlying disease, such as high blood pressure, diabetes, asthma, kidney failure, or tuberculosis; 91 (7.9%) had experienced self-isolation due to COVID-19; 174 (15.2%) had experience with COVID-19 testing; 4 (0.3%) were confirmed COVID-19 cases; 78 (6.8%) had a family member or friend with COVID-19; 362 (31.5%) reported that they had jobs that were easily exposed to COVID-19; and 889 (77.4%) thought that their company was satisfactorily dealing with COVID-19 quarantine measures. Finally, 219 (19.1%) of the respondents had experience using COVID-19–related apps.

### Intention to Use COVID-19–Related Apps

**Figure 1:**
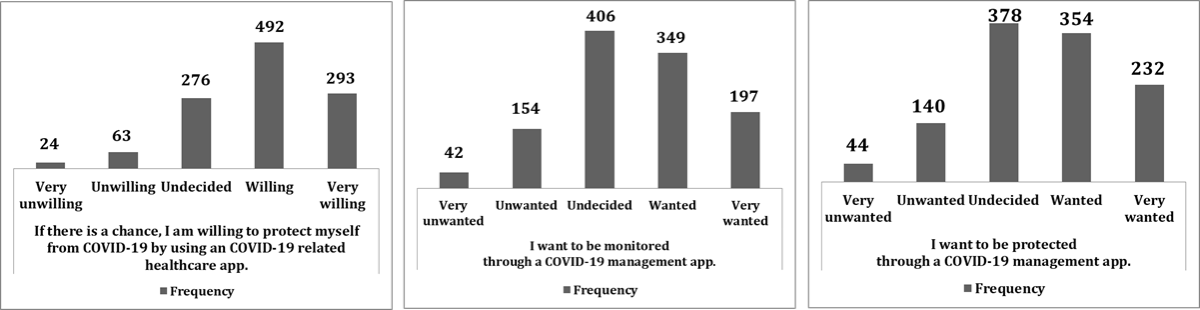
Intention to use COVID-19–related apps among survey respondents (N=1148).

This study assessed participants’ willingness to use COVID-19–related apps as shown in Figure 1. The first question asked the 1148 respondents if they were willing to protect themselves from COVID-19 by using COVID-19–related health care apps (Table 3), to which 68.4% (n=785) reported that they were “willing” or “very willing.” The second question asked the respondents whether they wanted to be monitored through a COVID-19 management app, to with 47.6% (n=546) of the respondents reporting that they wanted to be monitored. However, 35.4% (n=406) of the respondents had a neutral opinion about this. The last question asked respondents if they wanted to be protected through a COVID-19 management app; 51% (n=586) of the respondents wanted to be protected through a COVID-19 management app, whereas 32.9% (n=378) had a neutral opinion.

**Table 3:** Respondents’ (N=1148) intention to use COVID-19–related apps and the epidemiological investigation app.

Questions and intention to use the app			Participants, n (%)
**COVID-19–related apps**			
	**If one is available, I am willing to protect myself from COVID-19 by using a COVID-19–related health care app.**		
		Very unwilling	24 (2.1)
		Unwilling	63 (5.5)
		Undecided	276 (24.0)
		Willing	492 (42.9)
		Very willing	293 (25.5)
	**I want to be monitored through a COVID-19 management app.**		
		Very unwanted	42 (3.7)
		Unwanted	154 (13.4)
		Undecided	406 (35.4)
		Wanted	349 (30.4)
		Very wanted	197 (17.2)
	**I want to be protected through a COVID-19 management app.**		
		Very unwanted	44 (3.8)
		Unwanted	140 (12.2)
		Undecided	378 (32.9)
		Wanted	354 (30.8)
		Very wanted	232 (20.2)
**Epidemiological investigation app**			
	**Are you willing to use the epidemiological investigation app?**		
		Very unwilling	19 (1.7)
		Unwilling	41 (3.6)
		Undecided	238 (20.7)
		Willing	554 (48.3)
		Very willing	296 (25.8)

### App-Based Services to Support Activities to Overcome COVID-19

We surveyed which app-based services were needed to support activities aimed at overcoming COVID-19; this was a multiple-response question. Of the 1148 respondents, 709 (61.8%) reported that the epidemiological investigation app was the most necessary service (Table 4). In addition, respondents stated that a self-management app for self-isolation (613/1148, 53.4%), preventive self-route management app (605/1148, 52.7%), COVID-19 symptom management app (483/1148, 42.1%), and COVID-19–related information provision app (339/1148, 29.5%) were needed. The lowest percentage of responses (270/1148, 23.5%) received were regarding the use of mental health management apps.

**Table 4:** App-based services needed to support activities to overcome COVID-19.

Question and responses		Value	
		Responses, n (%) (n=3019)	Participants, n (%) (N=1148)
**Which app-based services are needed to overcome COVID-19?**			
	Epidemiological investigation app	709 (23.5)	709 (61.8)
	Self-management app for self-isolation	613 (20.3)	613 (53.4)
	Self-route management app	605 (20)	605 (52.7)
	COVID-19 symptom management app	483 (16)	483 (42.1)
	COVID-19–related information provision app	339 (11.2)	339 (29.5)
	Mental health management apps	270 (8.9)	270 (23.5)

### Intention to Use and Reasons for Reluctance to Use the Epidemiological Investigations App

**Figure 2:**
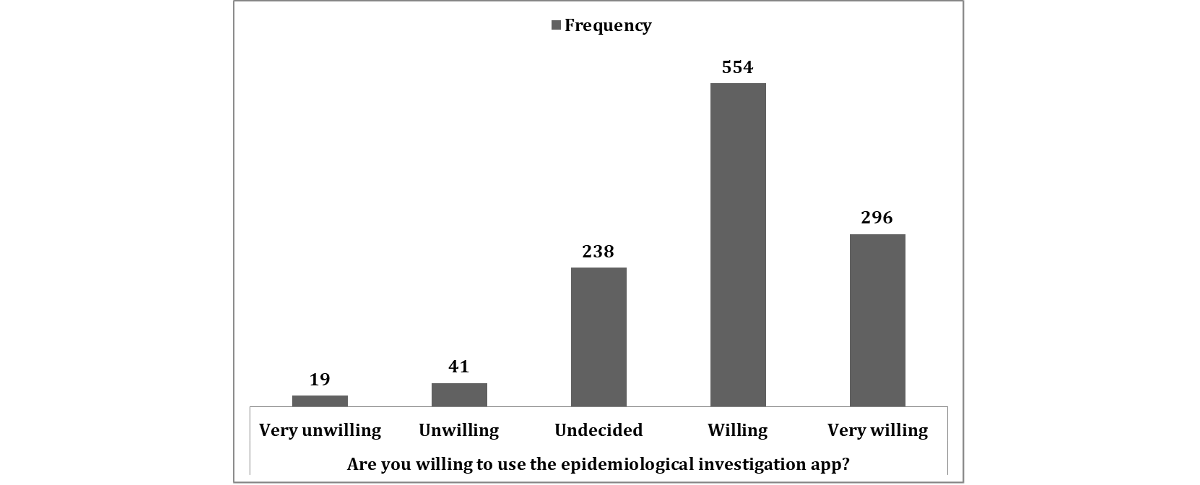
Willingness to use the epidemiological investigation app (N=1148).

First, we inquired whether the respondents were willing to use the epidemiological investigation app, which was reported as the most necessary service. In total, 554 of the 1148 (48.3%) respondents reported that they would use this app, with 296 (25.8%) indicating that they were very willing (Figure 2 and Table 3).

Second, we inquired why they were reluctant to use the app. Regarding this, of the 1148 respondents, 480 (41.8%) of respondents cited privacy concerns, 449 (39.1%) expressed concerns about personal information exposure and media disclosure, and 202 (17.6%) did not have a reason (Table 5). The response rate of those who were not reluctant was very low (13/1148, 1.1%).

**Table 5:** Reasons for reluctance to use the epidemiological investigation app (N=1148).

Question and responses		Participants, n (%)
**If you are reluctant to use the epidemiological investigation app, why?**		
	Privacy invasion problem	480 (41.8)
	Personal information exposure and media exposure	449 (39.1)
	Criticism and reproach of others	4 (0.3)
	Not reluctant	13 (1.1)
	No reason	202 (17.6)

### Intention to Use App-Based Services Required to Overcome the COVID-19 Crisis

The intention to use app-based services required to overcome COVID-19 were compared using the Wilcoxon rank sum test. The various app-based services evaluated in this study were as follows: (1) epidemiological investigation app, (2) self-management app for self-isolation, (3) self-route management app, and (4) COVID-19 symptom management app. The results indicated whether there were any differences in the intention to use these four apps according to the COVID-19–related characteristics of the respondents. These characteristics included the following: (1) presence of an underlying disease, (2) self-isolation experience, (3) COVID-19 test experience, (4) confirmed COVID-19 cases, (5) family members or friends with a confirmed COVID-19 case, (6) occupations that are easily exposed to COVID-19, (7) a company with good COVID-19 prevention strategies, and (8) experience with COVID-19–related apps (Table 6).

Regarding the presence of underlying disease and COVID-19–related app experience, there were significant differences in respondents’ intention to use the epidemiological investigation app, self-management app for self-isolation, self-route management app, and COVID-19 symptom management app. Moreover, those who had an underlying disease and had experience using COVID-19–related apps showed significantly higher intention to use these four apps (*P*=.05 and *P*=.01, respectively; Table 6).

**Table 6:** COVID-19–related characteristics and intention to use apps among survey participants (N=1148).

Variable and intention to use app		Participants, n (%)	Epidemiological investigation app		Self-management app for self-isolation		Self-route management app		COVID-19 symptom management app	
			Mean^a^ (SD)	*P* value	Mean (SD)	*P* value	Mean (SD)	*P* value	Mean (SD)	*P* value
**Presence of underlying disease**										
	No	1053 (91.7)	3.908 (0.871)	*.003* ^b^	3.944 (0.871)	.*03*	3.816 (0.900)	*.02*	3.885 (0.899)	*.04*
	Yes	95 (8.3)	4.168 (0.794)		4.158 (0.719)		4.021 (0.850)		4.095 (0.787)	
**Self-isolation experience**										
	No	1057 (92.1)	3.923 (0.868)	.28	3.953 (0.869)	.29	3.836 (0.898)	.65	3.900 (0.896)	.68
	Yes	91 (7.9)	4.000 (0.869)		4.066 (0.757)		3.791 (0.901)		3.934 (0.854)	
**COVID-19 test experience**										
	No	974 (84.8)	3.941 (0.842)	.75	3.977 (0.841)	.41	3.857 (0.857)	.27	3.912 (0.868)	.97
	Yes	174 (15.2)	3.862 (0.999)		3.874 (0.965)		3.695 (1.088)		3.851 (1.020)	
**COVID-19 confirmed person**										
	No	1144 (99.7)	3.929 (0.868)	.96	3.960 (0.860)	.17	3.836 (0.895)	.12	3.904 (0.892)	.26
	Yes	4 (0.3)	4.000 (0.816)		4.500 (1.000)		3.000 (1.414)		3.500 (1.000)	
**Family or friends with confirmed COVID-19**										
	No	1070 (93.2)	3.937 (0.851)	.72	3.959 (0.858)	.49	3.823 (0.900)	.15	3.901 (0.891)	.71
	Yes	78 (6.8)	3.821 (1.066)		4.000 (0.912)		3.962 (0.860)		3.923 (0.908)	
**Occupations that are easily exposed to COVID-19**										
	No	786 (68.5)	3.926 (0.852)	.55	3.961 (0.844)	.64	3.836 (0.867)	.63	3.912 (0.851)	.91
	Yes	362 (31.5)	3.936 (0.902)		3.964 (0.897)		3.826 (0.962)		3.881 (0.976)	
**A company with good COVID-19 prevention**										
	No	259 (22.6)	3.950 (0.841)	.74	4.042 (0.813)	.11	3.876 (0.797)	.58	3.969 (0.830)	.23
	Yes	889 (77.4)	3.924 (0.876)		3.938 (0.874)		3.820 (0.925)		3.883 (0.909)	
**COVID-19–related app experience**										
	No	929 (80.9)	3.896 (0.876)	*.004*	3.931 (0.877)	*.02*	3.799 (0.895)	*.002*	3.875 (0.896)	*.02*
	Yes	219 (19.1)	4.073 (0.815)		4.091 (0.779)		3.977 (0.896)		4.018 (0.867)	

^a^Respondents’ intention to use response values for each app, measured on a 5-point Likert scale, ranging from 1 = “very unwilling” to 5 = “very willing.”

^b^Italicized values indicate statistical significance.

## Discussion

This study aimed to determine the most essential apps required to overcome COVID-19 and the current status of the development of COVID-19–related apps in South Korea. Furthermore, this study aimed to determine users’ acceptance of and concerns related to the use of these apps.

First, respondents expressed a high level of willingness to use COVID-19–related apps. Many respondents indicated that they wanted to be protected and monitored by using COVID-19–related apps. However, many also had a neutral opinion. Thus, these apps need to be developed in a way to gain the trust of prospective users.

Second, the need to develop multiple apps emerged, which included epidemiological investigation apps, self-management apps for self-isolation, self-route management apps, COVID-19 symptom management apps (42%), and mental health management apps. Most of the respondents (61.8%) considered the epidemiological investigation app as the most needed app. In addition, the self-management app for self-isolation (53.4%), self-route management app (52.7%), COVID-19 symptom management app (42.1%), and mental health management app (23.5%) were marked as important, in that order.

In South Korea, there exists a self-management app for self-isolation, called “Self-quarantine Safety Protection App” [7,24]. However, based on the survey responses, it appears that various apps for self-management need to be developed. Regarding the self-route management app, apps using GPS and QR are increasingly being released; nevertheless, more apps are needed since their important continues to increase. To illustrate, the symptom management app helps identify new symptoms of COVID-19 and estimates the predicted value of specific symptoms [25]. In addition, these apps appear to be helpful in developing reliable screening tools. Thus, it is important to develop and utilize symptom management apps that can be used by the general public. Moreover, it was confirmed that there is also a demand for an app that can manage fatigue, mental health, and symptoms such as depression and anxiety caused by working from home and COVID-19 itself. There is ongoing research about COVID-19 survivors [26] and mental illness issues such as depression and anxiety caused by COVID-19 [27-29]. It has been reported that some people experienced worsened mental health after the pandemic [30]. This problem also applies to the medical staff such as physicians and nurses [31-33]. Therefore, active participation from the private sector and government is required to overcome the challenges posed by COVID-19. Furthermore, mental health problems need to be urgently addressed for groups such as COVID-19 survivors, medical staff, and women [29].

In the event of the COVID-19 pandemic, the main purpose of epidemiological investigations is to prevent early spread [34]; therefore, if the epidemiological investigation is delayed, secondary and tertiary disease transmission can occur, and people may become infected without knowing when or how they were infected. However, when a pandemic such as COVID-19 occurs, difficulties in epidemiological investigations and lack of workforce to conduct epidemiological investigation is often evident [35]. Therefore, there is also an urgent need to increase the number of epidemiological investigators, but this goal is difficult to achieve. To facilitate epidemiological investigations, a system that can actively cooperate with such investigations is needed. Consequently, if an epidemiological investigation app is developed, it can help actively provide basic information and medical records, including one’s own movements, at the time of confirmation by the epidemiological investigator [36]. However, respondents expressed concern about infringement of personal information used by these services, such as COVID-19 contact tracing apps based on GPS or smartphone logs. Therefore, like the epidemiological investigation app, a self-route management app is also needed to reduce the fear of personal information infringement and increase the amount of information provided for epidemiological investigations.

Third, the importance of privacy invasion issues of COVID-19–related apps was emphasized in this study. Despite the high intention to use the epidemiological investigation app, people were very concerned about privacy invasion issues, personal information exposure, and media exposure. Thus, it is vital to consider how to resolve people’s concerns about using these services, even after the necessary services are developed and available. Similarly, previous studies have found that people did not download and use contact tracing apps due to privacy concerns [37,38]. These findings suggest that it is important to design and develop apps deemed as necessary in order to overcome the COVID-19 crisis; however, to gain the public’s trust and make such apps available to many people, minimum amounts of personal information should be used and seek the public interest based on this. In this regard, a service where users have authority over their information should be developed [39].

Fourth, we found that those who had an underlying disease and had experience using COVID-19–related apps showed a significantly higher intention to use apps such as the epidemiological investigation app, self-management app for self-isolation, self-route management app, and COVID-19 symptom management app. Interestingly, even if users reported self-isolation experiences, COVID-19 test experiences, COVID-19 confirmed experiences, and nearby confirmed cases, their willingness to use COVID-19–related apps was not higher. In addition, no differences were found between the intention to use the apps among respondents engaged in occupations that had a relatively high exposure to COVID-19 cases or those employed by companies that complied with COVID-19 quarantine regulations. This is a surprising result; that is, the spread of COVID-19 is prevalent, but this does not directly lead to the use of apps. Hence, understanding people’s needs in the current situation is essential. The current findings suggest that a focus on promoting and distributing the service in view of the high intention of use by those with underlying diseases and those who have used COVID-19–related apps should be prioritized. In addition, the app should be promoted and distributed intensively in hospitals or health centers wherein people with underlying diseases may be easily accessible. It would also be beneficial to include a function that recommends other apps over existing apps so that various apps can be exposed to active prospective users. Furthermore, it should be considered that many people reported that they do want to use apps to overcome the COVID-19 pandemic.

COVID-19–related apps developed and used in South Korea ranged from those providing information to those used for symptom management, COVID-19 self-diagnosis, self-route management, mapping of COVID-19 cases, and reporting of COVID-19 confirmed cases. Based on the information needed at present, the COVID-19–related information app was found to be faithful to its function. As the COVID-19 pandemic continues, the development and use of GPS- and QR-based self-movement management and mapping of COVID-19 case services are needed. However, as mentioned earlier, these apps still have privacy issues. In addition, according to existing studies, the development ratio of Android- and iOS-based apps are similarly developed and used for COVID-19–related apps [12], but in South Korea, COVID-19 apps are mostly based on Android. Thus, a need to develop iOS-based apps for various Korean smartphone users is evident.

Despite the meaningful results discussed thus far, this study has several limitations. First, a total of 1148 survey respondents were analyzed. However, there were only four patients with confirmed COVID-19 among these participants, which is a low rate of 0.3% of the total respondents. Thus, to obtain more meaningful results, additional samples of COVID-19 confirmed cases should be collected. Second, although there are many COVID-19–related studies, there is limited published literature available. Third, 51.6% of the survey respondents were employed in white-collar jobs and managerial positions. Thus, occupational biases might have influenced the interpretation of results. Finally, to determine the current status of apps developed in South Korea, we conducted a search on application software downloading services. Two medical informatics professors and two researchers manually organized the app features in four meetings. To improve on this method, future research should apply tools to investigate the apps instead.

Despite these limitations, there are meaningful implications of the study’s findings. It was found that the COVID-19 apps may support activities aimed at overcoming the COVID-19 pandemic. However, our findings emphasized how several actions and requirements are necessary to accomplish this aim. Our findings further identified the most essential apps, as well as provided future directions for app development to overcome COVID-19. This study also emphasized the need for information protection to guarantee maximum privacy for users, thus increasing the likelihood of more users. Overall, several insights into the development of apps related to COVID-19 were identified, which can be utilized in future developments and improvements of new and existing apps related to COVID-19.

## Appendix

Multimedia Appendix 1 COVID-19-related apps in South Korea.

## Abbreviations

NRF: National Research Foundation of Korea
