# Supplementary material for: Development, Acceptance, and Concerns Surrounding App-Based Services to Overcome the COVID-19 Outbreak in South Korea: Web-Based Survey Study
Source: JMIR Med Inform. 2021 Jul 30;9(7):e29315. doi: 10.2196/29315 (PMC8330629; doi:10.2196/29315)
Supplement: Multimedia Appendix 1 [file medinform_v9i7e29315_app1.docx]

**Appendix**

Table S1. COVID-19-related apps in South Korea (2021.3)

| **No** | **App** | **App** | **OS** | **Main features of the app** |
| --- | --- | --- | --- | --- |
|  |  |  |  |  |
| 1 | CORNANOW  **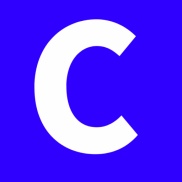** | **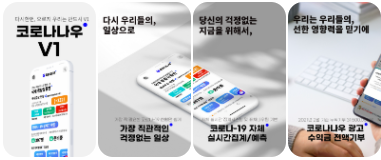** | Android  Website | - Release date: 2020. 2  - Provider: rdod205  - Contents: COVID-19 status, mask information, COVID-19 related news, screening clinic information, etc. |
| 2 | 코로나 탐색기  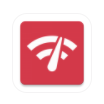 | **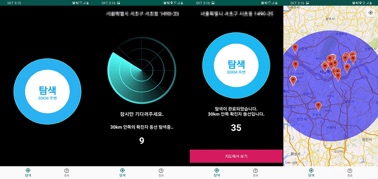** | Android | - Release date: 2020. 2. 7  - Provider: venusblessing  - Contents: Checking the confirmed person of COVID-19 near the user. |
| 3 | 코로나앱  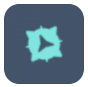 | **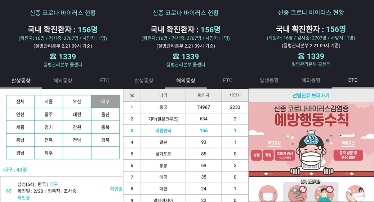** | Android | - Release date: 2020. 2. 17  - Provider: 은갱스  - Contents: COVID-19 status, worldwide COVID-19 status |
| 4 | 코로나 접촉검사  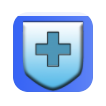 | **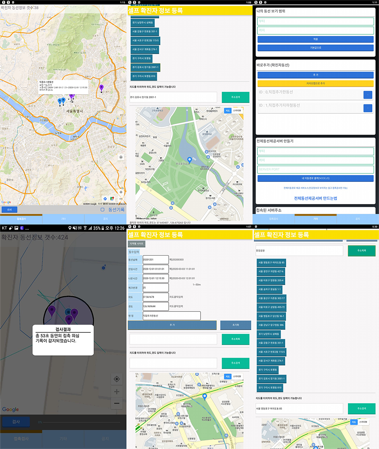** | Android | - Release date: 2020. 2. 25  - Provider: kruzfactory  - Contents: Users themselves compare their movements in the past with to COVID-19 patient’s movements |
| 5 | 코로나19 상황판 실시간  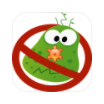 | **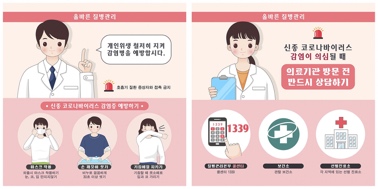** | Android | - Release date: 2020. 2. 26  - Provider: streampod  - Contents: COVID-19 related real-time situation board |
| 6 | 코로나19 현황판  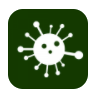 | **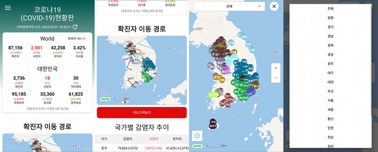** | Android | - Release date: 2020. 3. 2  - Provider: escnet  - Contents: Real-time situation board related to COVID-19 |
| 7 | 코로나19 경남  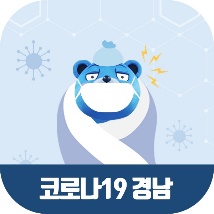 | **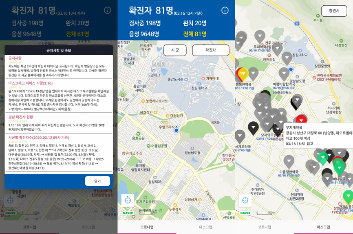** | Android | - Release date: 2020. 3. 6  - Provider: wosung102  - Contents: Gyeongsangnam-do COVID-19 confirmed route, public mask inventory Notification, public mask inventory notification |
| 8 | 코로나침반  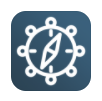 | **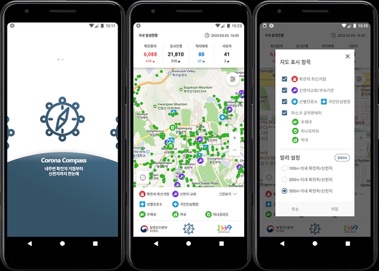** | Android | - Release date: 2020. 3. 6  - Provider: spiderman  - Contents: Location and phone number of the screening center,  location of the National Safety Hospital and availability of outclinic or hospitalization, notification when COVID-19 patient or Shincheonji Church is close to the user. |
| 9 | 코로나맵  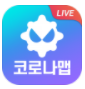 | **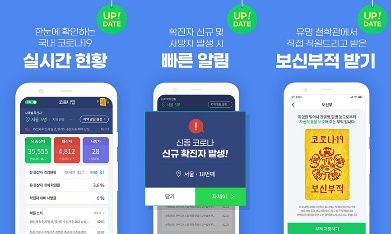** | Android | - Release date: 2020. 3. 6  - Provider: coreplanet  - Contents: COVID-19 status, confirmed person route, Screening Clinic |
| 10 | 웨어마스크  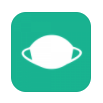 | **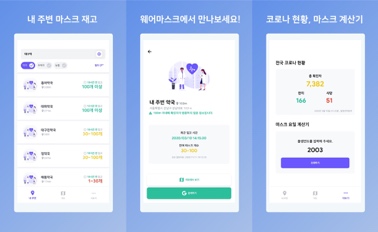** | Android | - Release date: 2020. 3. 9  - Provider: 정찬효  - Contents: Real-time notification of public mask inventory and incoming time near user |
| 11 | 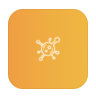코로나19 소식전달 | **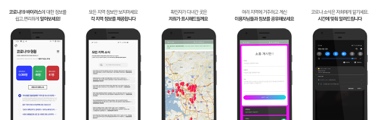** | Android | - Release date: 2020. 3. 9  - Provider: Novus3P  - Contents: COVID-19 related information notification, communication bulletin board for users living in the same area |
| 12 | 코로나핀  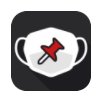 | **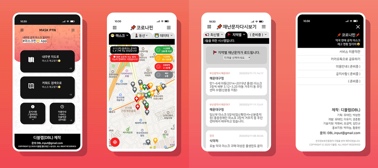** | Android | - Release date: 2020. 3. 10  - Provider: DBL  - Contents: Find the public mask, Notify the mask, COVID-19 situation board, Replay the disaster text. |
| 13 | 코로나가  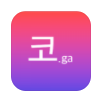 | **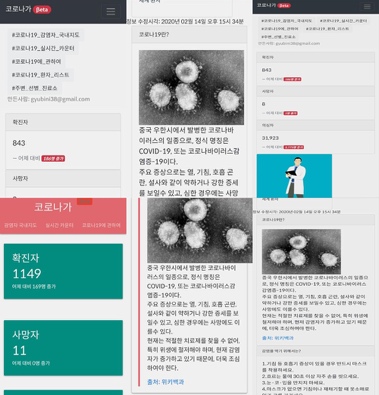** | Android | - Release date: 2020. 3. 11  - Provider: redev S  - Contents: Check the COVID-19 confirmed people and their movements. |
| 14 | NEAR  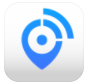 | **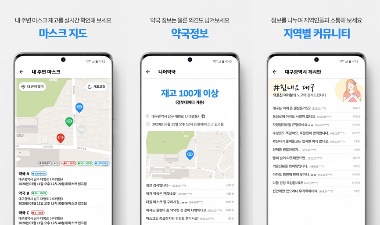** | Android | - Release date: 2020. 3. 11  - Provider: Tag corporation  - Contents: Pharmacy information, mask inventory, voice support service for the visually impaired |
| 15 | 코로나맵 위키 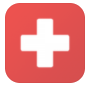 | **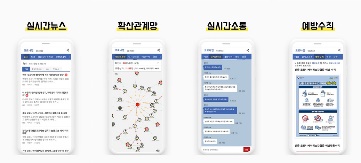** | Android | - Release date: 2020. 3. 12  - Provider: coronamap  - Contents: Information on the status of confirmed cases, movement of confirmed cases, news, mask inventory status, and prevention information |
| 16 | 당근마스크  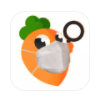 | **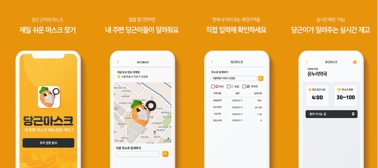** | Android | - Release date: 2020. 3. 12  - Provider: softpower  - Contents: Real-time mask inventory check, guide to mask sales location near user |
| 17 | MARK  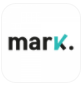 | **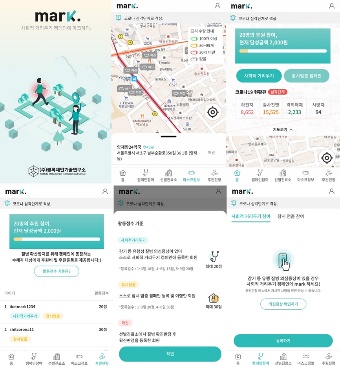** | Android | - Release date: 2020. 3. 18  - Provider: dev@ibct.kr  - Contents: Mask purchase information, COVID-19 status, screening clinic, sponsorship function |
| 18 | 코백플러스  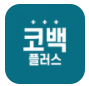 | **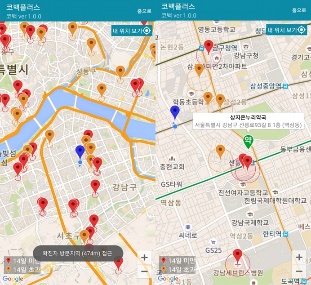** | Android | - Release date: 2020. 3. 18  - Provider: tina3d.ent  - Contents: Confirmed person movement, public mask purchase information, worldwide COVID-19 status |
| 19 | 마스크어딨니  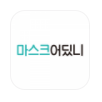 | **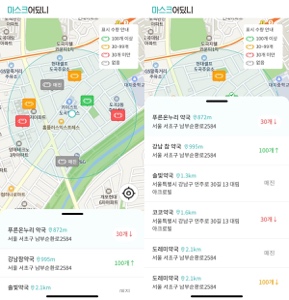** | Android | - Release date: 2020. 3. 20  - Provider: dev@ibct.kr  - Contents: Public mask store and inventory status near user |
| 20 | 마스크타임  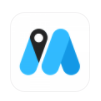 | **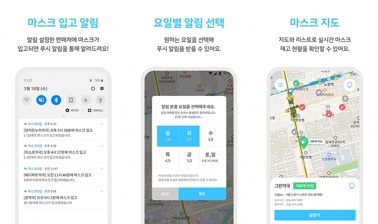** | Android | - Release date: 2020. 3. 20  - Provider: Masktime  - Contents: Set up notifications related to public mask stores and provide push notifications when masks are in stock |
| 21 | 알려줘  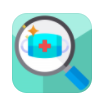 | **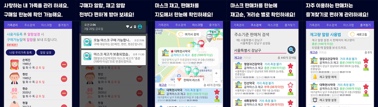** | Android | - Release date: 2020. 3. 20  - Provider: ados397  - Contents: Provides a variety of information about the masks that are most needed in the Pandemic. |
| 22 | 코로나19  자가진단  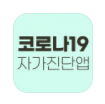 | **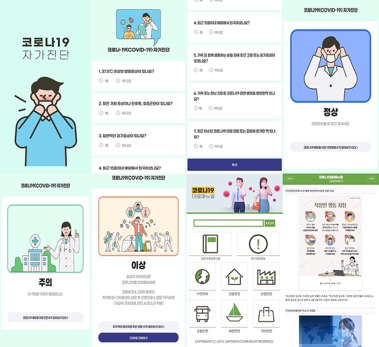** | Android | - Release date: 2020. 3. 30  - Provider: JINOSYS  - Contents: COVID-19 Self-Diagnosis App |
| 23 | BMC 코로나19  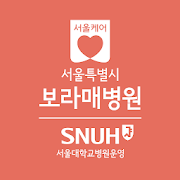직원지킴이 | **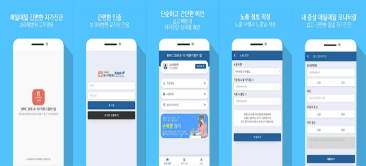** | Android  iOS | - Release date: 2020. 4  - Provider: Seoul national Univ. boramae medical center  - COVID-19 self-test symptoms for employees, report symptom employee occurrence alarm |
| 24 | 코로나 지침 검색  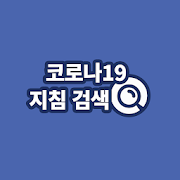 | 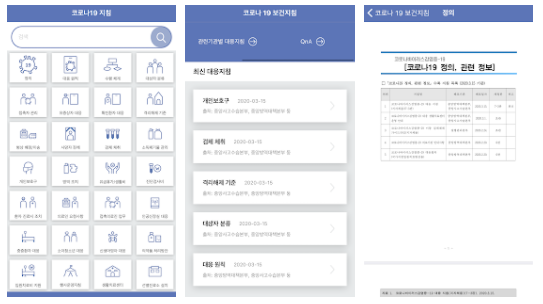 | Android  iOS | - Release date: 2020. 4.  - Provider: Seoul Metropolitan Government  - Contents: COVID-19 guidelines |
| 25 | 코로나월드  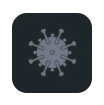 | **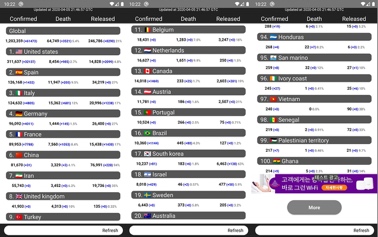** | Android | - Release date: 2020. 4. 6  - Provider: reiui9  - Contents: It marks the progress of COVID-19 confirmed patients around the world. |
| 26 | JINOSYS  전자출입명부  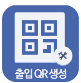 | 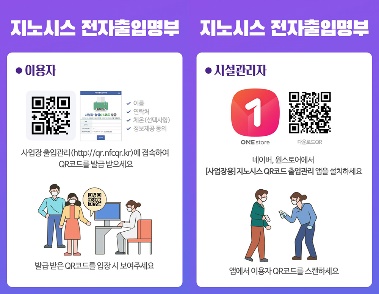 |  | - Release date: 2020.5.22  - Provider: JINOSYS |
| 27 | Incheon 코로나19  꼼짝 마!  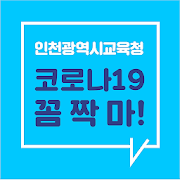 | **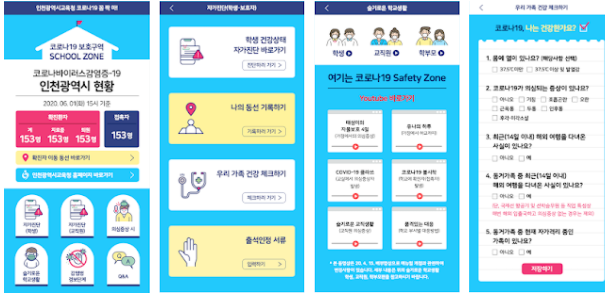** | Android  iOS | - Release date: 2020. 6.  -Provider: Incheon education science & informatics institute  - Contents: Infectious disease symptom management, infectious disease-related information provision service, self-movement management for prevention |
| 28 | 학교 안전지킴이  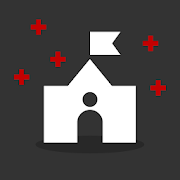 | **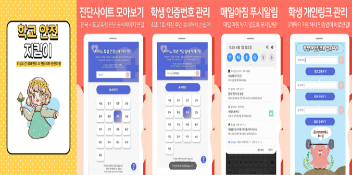** | Android | - Release date: 2020. 5. 21  -Provider: 앱만드는선생님  - Contents: Infectious disease symptom management, search for nearby emergency hospital |
| 29 | 코로나메모  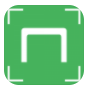 | **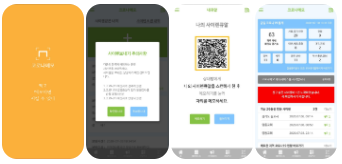** | Android | - Release date: 2020. 7. 13  -Provider: Tablemedia  - Contents: Memo of place of visit using QR code, mapping of confirmed person's movement, COVID-19 status |
| 30 | 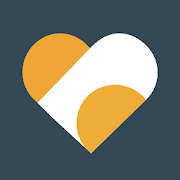FAMY 2.0 | 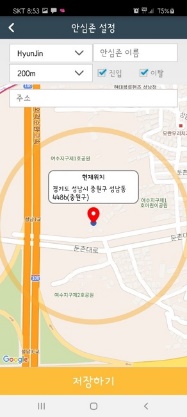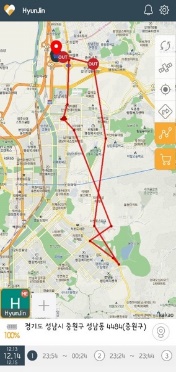 | Android | - Release date: 2020. 8.  - Provider: Gper  - Contents: User's daily route recording, safe zone setting, Kakao Navi linkage, indication of risk areas for confirmed cases |
| 31 | 코로나지수  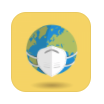 | **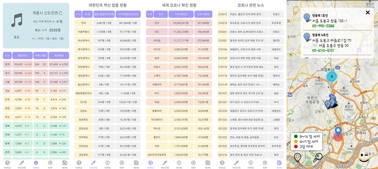** | Android | - Release date: 2020. 8. 7  - Provider: goesnow  - Contents: New COVID-19 confirmed patient information can be checked every day. |
| 32 | 코로나 패스  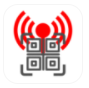 | 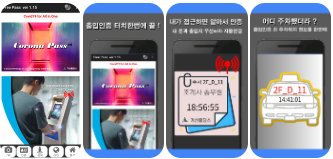 | Android | - Release date: 2020. 08. 10  - Provider: sky2711  - Contents: Location storage system via GPS and NFC |
| 33 | 코로나 탐지기  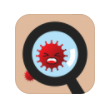 | **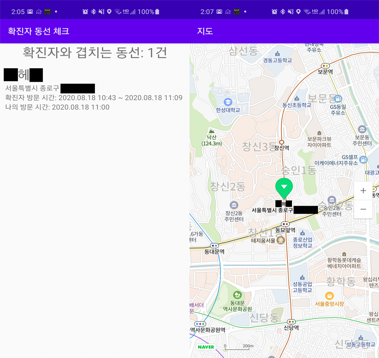** | Android | - Release date: 2020. 8. 31  - Provider: mg2000  - Contents: Compare the movement of the user with the movement of the COVID-19 patients to determine whether their movements overlap or not. |
| 34 | KFKOREA  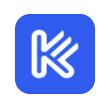 | **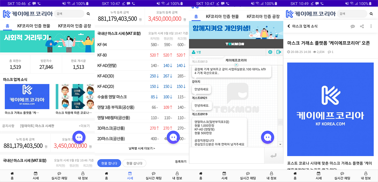** | Android | - Release date: 2020. 9. 8  - Provider: yay.waieiwai  - Contents: Mask market application |
| 35 | 코로나  위치추적  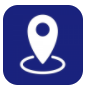 | **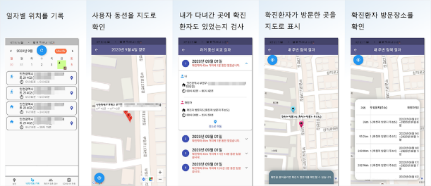** | Android | - Release date: 2020. 9. 11  - Providers: JungHoon Lee  - Contents: Location record, user's movement, e-confirmation visited place indication and redundancy |
| 36 | 코로나피하go  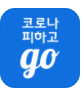 | **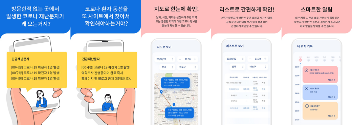** | Android | - Release date: 2020. 10. 6  - Provider: 2gather  - Contents: Infectious disease information, self-directed movement management for prevention, confirmation of the confirmed person's movement |
| 37 | Koala  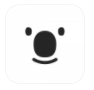 | **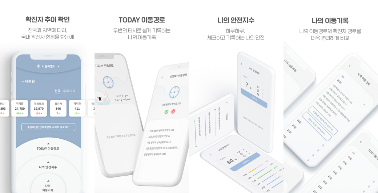** | Android | - Release date: 2020. 10. 12  - Provider: Eucalyptus  - Contents: COVID-19 confirmed person information, route record, confirmed person route, user safety index |
| 38 | 코로나알리미  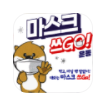 | **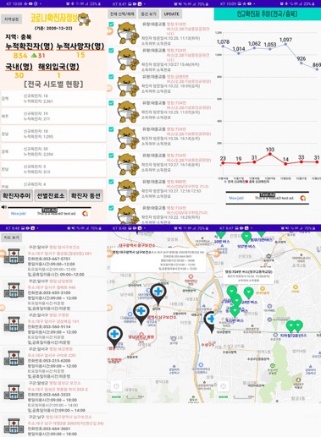** | Android | - Release date: 2020. 12. 1  - Provider: dbstj76  - Contents: Real-time status of patient of COVID-19, COVID-19 Information Guide of Daegu City, South Korea. |
| 39 | COVID SHIELD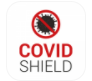 | **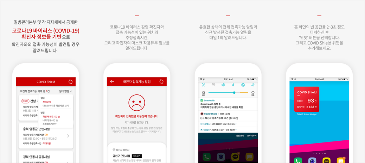** | Android | - Release date: 2020. 12. 11  - Provider: mobilefactory  - Contents: Notification of possibility of contact with confirmed person |
| 40 | Corona Safer  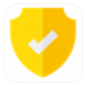 | **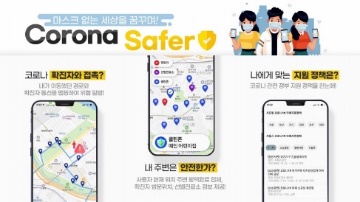** | Android | - Release date: 2020. 12. 23  - Providers: coronasafer  - Content: User route record, user movement line and confirmed person movement line mapping, Records of risk, nearby screening clinics, and safe places |
| 41 | Hanyang Univ.  코로나 접촉  추적앱  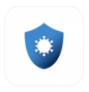 | **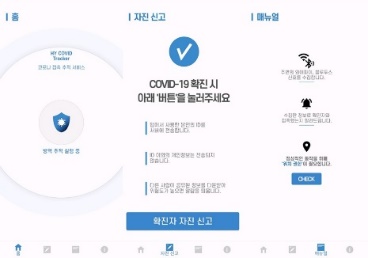** | Android | - Release date: 2021. 1. 6  - Providers: kty0264  - Contents: Preventive self-movement management, epidemiological investigation of confirmed cases, voluntarily reports the COVID-19 case |
| 42 | 코로나 신호등  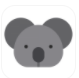 | **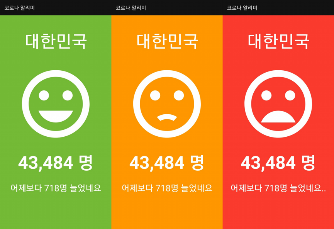** | Android | - Release date: 2021. 1. 28  - Provider: junsung0713  - Contents: Information on the number of COVID-19 confirmed patients in color and emoticon |
| 43 | 코현모  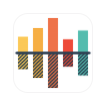 | **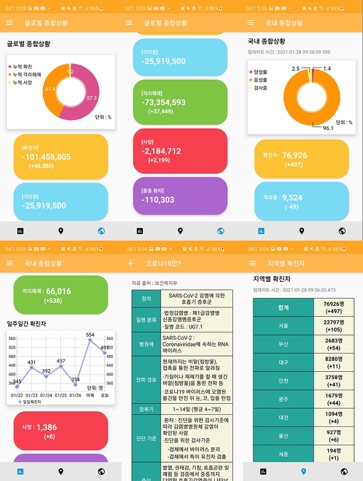** | Android | - Release date: 2021. 2. 8  - Provider: 박상우  - Contents: COVID-19 status data is updated and provided every day. |
| 44 | 코로나19  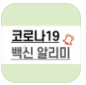백신알리미 | **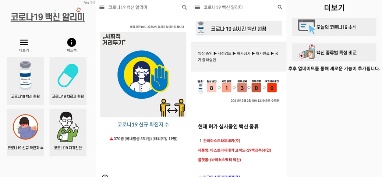** | Android | - Release date: 2021. 2. 3  - Provider: 이선권  - Contents: Vaccine status, status of confirmed cases, news, symptom management, self-diagnosis |
| 45 | 코로나  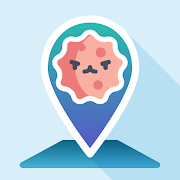동선안심이 | **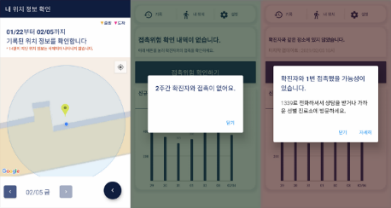** | Android  iOS | - Release date: 2021. 2. 9  - Provider: cryptolab  - Contents: Notification of occurrence of risk of contact with confirmed patients, location-based service, presence of risk of contact with confirmed patients |
| 46 | 코로나돋보기  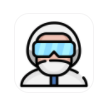 | **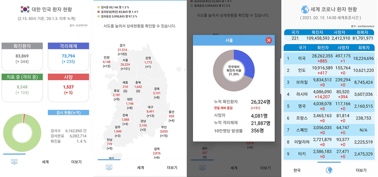** | Android | - Release date: 2021. 2. 16  - Provider: HLB  - Contents: Provide information on the current status of COVID-19 in Korea and around the world. |
| 47 | 코로나지겹다  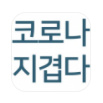 | **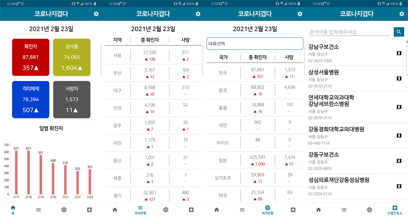** | Android | - Release date: 2021. 3. 3  - Provider: 코지개  - Contents: It provides the status of COVID-19 infection in Korea and around the world. |
| 48 | 코브리움  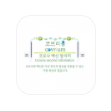 | **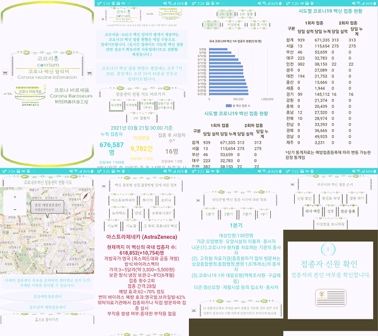** | Android | - Release date: 2021. 3. 22  - Provider: 김건재  - Contents: COVID-19 vaccine status in Korea and around the world, other information on the COVID-19 vaccine. |
